# *Xylosandrus crassiusculus* (Motschulsky) on Cocoa Pods (*Theobroma cacao* L.): Matter of Bugs and Fungi

**DOI:** 10.3390/insects13090809

**Published:** 2022-09-05

**Authors:** Shivaji Hausrao Thube, R. Thava Prakasa Pandian, Arulappan Josephrajkumar, Anthara Bhavishya, B. J. Nirmal Kumar, Dnyaneshwar M. Firake, Vivek Shah, T. N. Madhu, Enrico Ruzzier

**Affiliations:** 1 ICAR—Central Plantation Crops Research Institute, Regional Station, Vittal 574243, Karnataka, India; 2 ICAR—Central Institute for Cotton Research, Nagpur 440010, Maharashtra, India; 3 ICAR—Central Plantation Crops Research Institute, Regional Station, Kayamkulam 690533, Kerala, India; 4 ICAR—Directorate of Floricultural Research, Pune 411005, Maharashtra, India; 5 World Biodiversity Association Onlus, c/o Museo Civico di Storia Naturale Lungadige, Porta Vittoria 9, 37129 Verona, Italy

**Keywords:** ambrosia beetle, symbiosis, fungus, yeast, *COI*, tea mosquito bug

## Abstract

**Simple Summary:**

During standard monitoring activities aimed at preventing the insurgence of a pest infestation in a cocoa plantation in Karnataka (India), a sudden and unprecedented infestation of cocoa pods was recorded in 2021. The symptoms were the exudation of a brownish gummy substance from mature pods and the presence of pinhead-sized holes on the pod husk. Morphological and molecular characterization identified the cause of the symptoms to be the activity of the native ambrosia beetle *Xylosandrus crassiusculus.* Given the uniqueness of the attack, research was undertaken to investigate the causes of the infestation, describe the type of damage caused, and characterize possible fungal symbionts that allow the beetle to reproduce. Results showed that the infestation of *X. crassiusculus* was most likely triggered by previous attacks of *Helopeltis theivora* (Hemiptera: Miridae), a common pest that causes necrotic injuries to cocoa pods. Furthermore, the success of *X. crassiusculus* using cocoa as a reproductive substrate appeared to be dependent on its symbiotic fungi and yeasts, possibly involved in the detoxification or enrichment of the substrate, besides being the food source of the beetle.

**Abstract:**

Exudation of mucilage from pinhead-sized boreholes in cocoa pods was recorded in Karnataka, India, during 2021. Further investigations showed the association of scolytine beetles with infested pods. The identity of the pest, *Xylosandrus crassiusculus*, was confirmed through morphological characterization and sequencing of the mitochondrial *COI* gene. We studied the predisposing factors for its infestation, visible and concealed damaging symptoms, and fungal symbionts. In addition to its well-known symbiotic fungus, *Ambrosiella roeperi*, a new association of yeast, *Ambrosiozyma monospora,* was discovered. We also traced the possible role of the mirid bug, *Helopeltis theivora,* in host selection by *X. crassiusculus*. Overall results indicated that a ‘mirid bug-ambrosia beetle–pathogen complex’ is responsible for the severe damage to cocoa pods in South India.

## 1. Introduction

Cocoa (*Theobroma cacao*: Malvaceae) is an important crop native to South and Central America. It is widely cultivated in several humid tropical countries around the globe [1]. In India, cocoa plantations cover approximately 97,500 hectares and produce almost 25,800 million tons per year [2]. In India, cocoa is mainly grown in Kerala, Karnataka, Tamil Nadu, and Andhra Pradesh, with the latter contributing to approximately 40% of the country’s total production [2,3]. India exported more than 15,000 million tons of cocoa products during 2013 and 2014 [4,5].

As an exotic crop in most global production areas, the plant is susceptible to several diseases and pests, which affect its vitality and productivity or compromise the marketability of the cocoa bean. Furthermore, through increasing global trade and transport of planting material, pests and diseases originating from the native range of *T. cacao* are now being dispersed all over the world. Specifically, insect pests are one of the key factors affecting the production of cocoa in India [6]. All these situations pose important challenges for cocoa production, which is currently oriented towards sustainability and reduced environmental impact.

Among the more than fifty insect pests affecting cocoa [7], the tea mosquito bug (TMB) species complex (*Helopeltis theivora* Waterhouse, 1886, *H. bradyi* Waterhouse, 1886 and *H. antonii* V. Signoret, 1858) (Hemiptera: Miridae) is considered one of the most significant threats to cocoa cultivation across the globe [8]. TMB, and *H. theivora* especially, has emerged as a major pest of cocoa plantations in South India, causing up to 40% yield loss [9,10,11]. Both nymphs and adults puncture plant parts such as leaves, shoots, peduncles, and pods to suck plant sap, leading to gum exudation from the punctures [12]. Furthermore, the punctures cause circular tissue necrotization, damage that allows the settlement of secondary plant pathogens [12].

Wood boring beetles (Coleoptera: Curculionidae: Scolytinae) are another group of insects capable of damaging cocoa plantations [13]. Most of these beetles are not primary pests because they target stressed or decaying trees [14]. However, their damage is conspicuous. The beetles excavate tunnels in the sapwood in which they cultivate their symbiotic fungi that act as a food source for the adults and larvae. Some species, though, are major pests of fruit crops, including the coffee berry borer (*Hypothenemus hampei* (Ferrari, 1867)), and others such as *Hypothenemus crudiae* (Panzer, 1791), *Hypothenemus obscurus* (Fabricius 1801), and *Hypothenemus seriatus* (Eichhoff, 1872) have been reported as damaging and/or breeding in cocoa plantations [15].

In September 2021, a sudden and unusual oozing of a brownish gummy substance from the pods of a 14-year-old cocoa plantation was observed in Karnataka, India. A preliminary investigation showed that the cause of the gum emission was the boring activity of a scolytine beetle. Furthermore, field observations indicated that cocoa fruits previously damaged by *H. theivora* were the target of scolytine beetles.

Given the uniqueness of the discovery and the possible phytosanitary relevance of such infestations, an investigation was conducted: i. to identify the scolytine species boring into the cocoa pods; ii. to describe the type of damage caused by the beetle; iii. to evaluate the possible co-occurrence of plant pathogens; and iv. to investigate the factors causing the infestation, specifically, the role of *H. theivora* triggering the scolytine beetle attack.

## 2. Materials and Methods

### 2.1. Study Site, Sample Collection, and Pest Incidence

Damaged cocoa pods were collected from a 15-year-old plantation (Upper Amazon cocoa genotypes) located at the Indian Council of Agricultural Research—Central Plantation Crops, Regional Station, Vittal (ICAR-CPCRI-RS, Vittal), Karnataka, India (12.45° N, 75.4° E; 90 MASL) during the first week of September 2021. The cocoa grafts were planted as a mixed crop at a spacing of 2.7 m × 5.4 m during 2007, under 2.7 m × 2.7 m spaced areca nut palms. Pruning of the cocoa was performed in August–September to give an umbrella shape to the crop. The pruned biomass was used for mulching the basin after the nutrient application. About 20 L of water were supplied to each tree in a span of 24 h through a drip irrigation system. The recommended dosage of fertilizers (100:18:117 NPK g/tree) was applied in two equal splits during the last week of May and October in the form of urea (220 g), rock phosphate (200 g), and muriate of potash (235 g) as sources of N, P, and K fertilizers. Fertilizer was applied around the tree at a distance of 75 cm away from the main stem and mixed with the soil by raking.

Infested pods were examined in the laboratory, and the insect specimens were recovered from cocoa beans. Pest severity (number of insect-pests per pods, *n* = 20) and percent of pod infestation were determined for a 0.40-hectare cocoa plantation. Subsequently, different cocoa-growing areas were surveyed in the Dakshina Kannada district of Karnataka, India, to assess the distribution pattern/range of this pest (Supplementary Table S1).

### 2.2. Insect Identification

Adult beetles were morphologically identified using available identification keys [16]. Four adult females, randomly selected after morphological identification, were used for amplification and sequencing of the *mt-COI* gene. The procedure for DNA extraction, PCR, and sequencing followed Thube et al. [17,18]. The amplified product was separated with electrophoresis (Best Lab International Inc., China) using a 1.0% agarose gel [19]. PCR purification (Geneaid, Taiwan) was performed, and the purified products were sent for Sanger sequencing (Biokart India Pvt. Ltd., India). Obtained sequences were aligned using BioEdit (Biological sequence alignment editor—Tom Hall, http://www.mbio.ncsu.edu/BioEdit/bioedit.html, accessed on 15 January 2022) and blasted in the NCBI (http://www.ncbi.nlm.nih.gov/, accessed on 27 January 2022) as well as the BOLD database (http://www.boldsystems.org/, accessed on 27 January 2022). Sequence similarity was analyzed with the available DNA sequences in GenBank, and sequences were deposited in NCBI.

### 2.3. Documentation of Symptoms and Gallery Measurements

All the visible damage symptoms of the pest were recorded by observing the infested pods (*n* = 25) directly in the field. However, all the concealed damage symptoms (appearance of galleries inside pod husk, mucilage, and beans, and the location of immature stages of the pest within the infested pods) were systematically recorded after cutting open the infested pods (*n* = 20) in the laboratory. The length and width of the insect galleries (*n* = 20) from infested pods were measured using a stereomicroscope (Leica M10 equipped with an EC4 Digital camera).

### 2.4. Isolation and Identification of Associated Fungi

**Figure 1:**
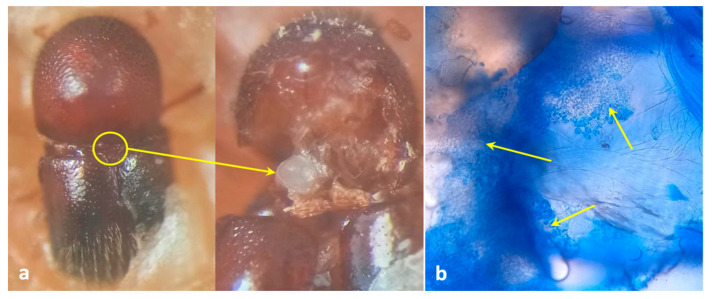
Mycangial dissection. (**a**) Location and view of mycangia; (**b**) view of symbionts harboring mycangia after staining with lactophenol cotton blue (arrows indicate the presence of fungal propagules inside mycangia).

Mycangia of adult females (*n* = 10) were dissected (Figure 1) after surface sterilization with a 1% sodium hypochlorite solution for 10 s. Dissected mycangia were pinpricked using a sterile needle, suspended in 500 μL sterile distilled water, and vortexed for 30 s. About 50 μL of this suspension was inoculated separately on a potato dextrose agar (PDA) medium amended with rifampicin (100 mg/mL) as an antibiotic. Simultaneously, beetle galleries (*n* = 20) containing fungal propagules were inoculated separately on PDA media and incubated at 28 ± 2 °C for 7–10 days. Here, we focused on the primary fungi associated with the ambrosia beetle, so only the fungi present in both the beetle mycangia and galleries were selected and described [20].

Symbiotic fungi isolated from mycangia and beetle galleries were initially identified using cultural colony and microscopic characterization [21,22]. The identity of symbiont isolates (except *Fusarium* sp.) was further confirmed by molecular characterization using amplification of ribosomal DNA following the methodology used in [18]. The fungal isolate belonging to genus *Fusarium* was analyzed using multigene amplification with the internal transcribed spacer region of ribosomal DNA [23], translation elongation factor 1α [24], and RNA polymerase II large subunit [24]. The amplified product was visualized using 1% agarose gel, purified using Geneaid PCR Purification Kit (Geneaid Biotech Ltd., New Taipei City, Taiwan), and the DNA fragment was sequenced by Sanger sequencing (Biokart India Pvt. Ltd., Bengaluru, India). The obtained sequences were aligned using BioEdit (Biological sequence alignment editor—Tom Hall, http://www.mbio.ncsu.edu/BioEdit/bioedit.html, accessed on 15 January 2022) and compared with the available sequences in NCBI (http://www.ncbi.nlm.nih.gov, accessed on 27 January 2022). The obtained sequences were deposited in NCBI.

### 2.5. Choice Test Assay against Healthy and Mirid Affected Cocoa Pods

A choice test assay [6] was conducted to determine the preference of the scolytine female specimens between healthy and mirid affected pods. Healthy and damaged cocoa pods of uniform size (400 g), age (110 days old pods), and variety (Upper Amazon collection) were harvested from the germplasm block of ICAR-CPCRI-RS, Vittal. Two pods from each category (healthy and mirid infested) were transferred to a single insect rearing cage (aluminum cage size: 15 × 15 × 20 cm; 18 gauge), and ten adults were released in each cage. This treatment was replicated six times and the entire experiment was repeated twice to obtain an affirmative result. Observations on the entry of adults inside the pods were recorded at the 24-h interval until the entry of all beetles into pods. All bored pods were cut open five days after beetle penetration to determine and evaluate the position of the insects throughout pod tissues.

## 3. Results

### 3.1. Insect Identification

**Figure 2:**
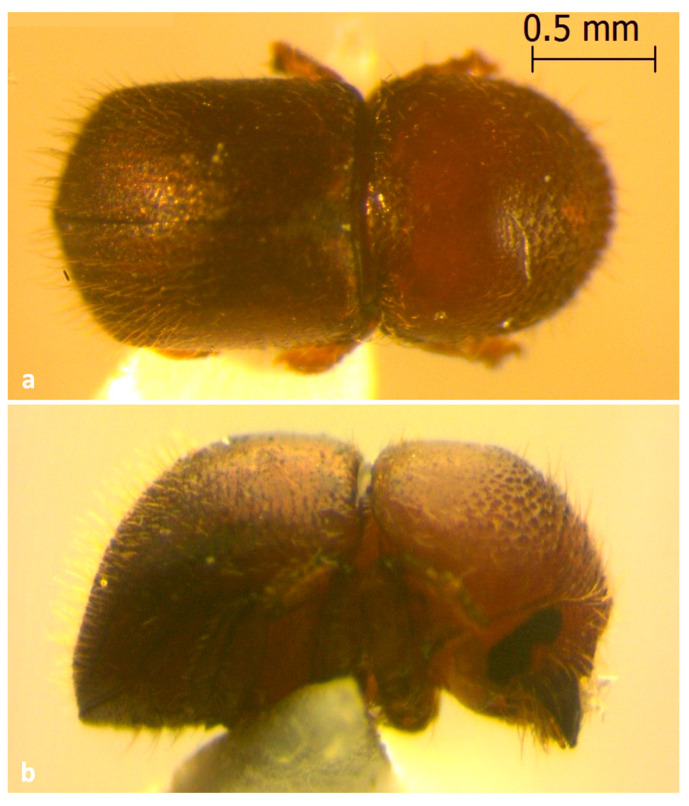
Adults of *Xylosandrus crassiusculus* recovered from cocoa pods. (**a**) Dorsal view; (**b**) Lateral view.

All collected beetles were morphologically identified as *Xylosandrus crassiusculus* (Motschulsky, 1866) (Figure 2a,b). The *mt COI* gene sequences (GenBank accession OM442994-OM442997) were 98.86% similar to *X. crassiusculus* from India (GenBank accession MZ895373).

### 3.2. Pest Incidence and Damaging Symptoms

**Figure 3:**
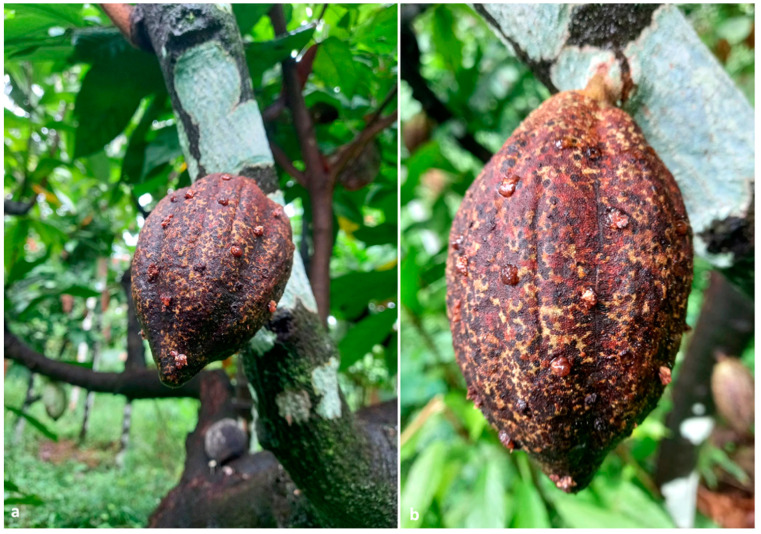
(**a**) Cocoa pod exhibiting oozing of brownish sap and frass; (**b**) detailed view of the attacked pod showing the scattered distribution of the boring points of the scolytine beetles.

**Figure 4:**
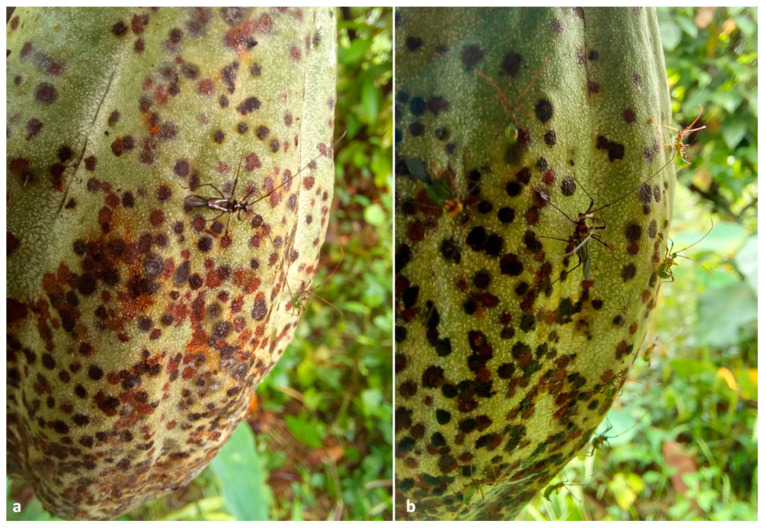
(**a**,**b**) Close views of cocoa pods attacked by *Helopeltis theivora*. The brownish circular spots are fruit necroses induced by the bug piercing with their mouthparts. In both images, *H. theivora* adults and nymphs can be observed while feeding upon the pod.

**Figure 5:**
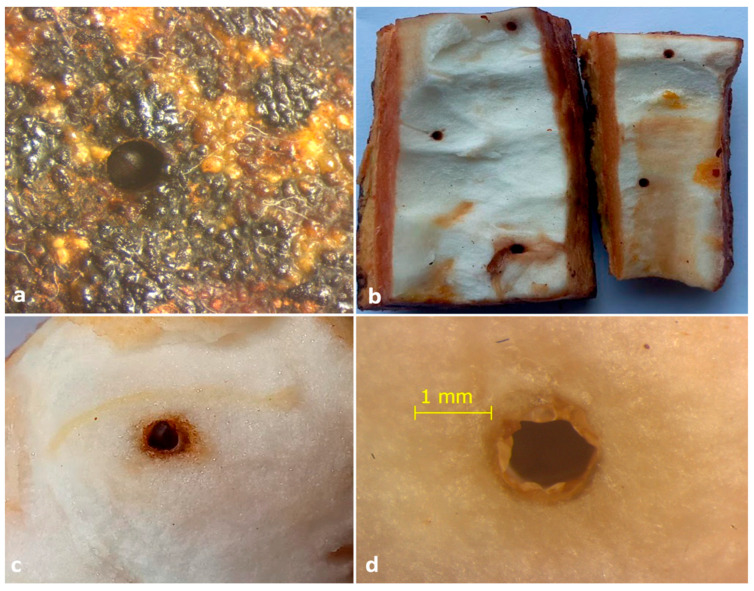
(**a**) Entrance hole of *X. crassiusculus*; beetle closing the entrance of the gallery with its elytral declivity visible; (**b**) view of the inside of the rind of a cocoa pod showing the entrance holes surrounded by brown tissue; (**c**) view of the gallery on the mucilage tissue of the fruit; (**d**) close view of the gallery showing the necrotic brown tissue.

**Figure 6:**
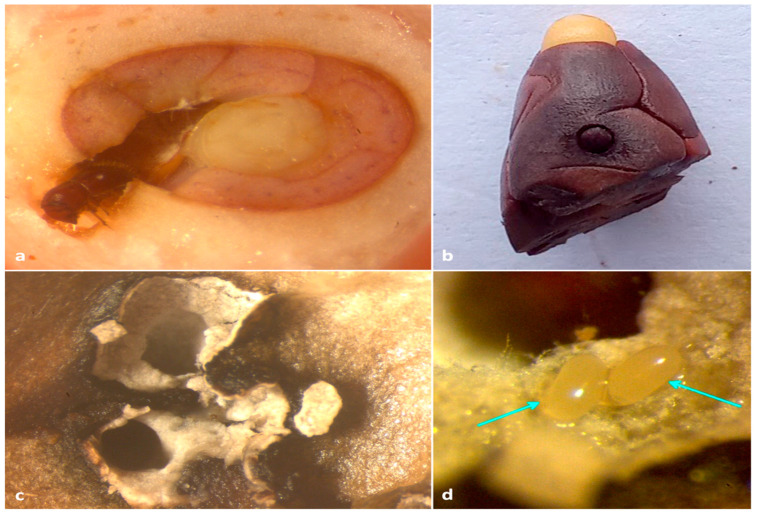
(**a**) adult *X. crassiusculus* boring on an immature cocoa bean; (**b**) adult *X. crassiusculus* tunneling on mature cocoa bean; (**c**) staining of insect galleries with whitish-grey fungal hyphae; (**d**) eggs of *X. crassiusculus* with fungal symbionts in cocoa bean.

In a 0.40 ha plantation located in Vittal, around 4.6% (12 out 260) of the cocoa trees were bearing 1.54% (150 out 9750) of infested pods. The number of adult beetles per infested pod ranged from five to 22, with a mean of 13.25 ± 1.21. Cocoa pods invaded by *X. crassiusculus* exhibited oozing of a brownish gummy substance (Figure 3a,b); furthermore, the outer husk of the pod was clearly covered with multiple circular necrotic spots caused by the feeding activity of *H. theivora* (Figure 4a,b). Small pinhead-sized holes were visible upon the removal of the gummy secretion (Figure 5a). Internal tissues surrounding the beetle galleries were yellowish-brown (Figure 5b,c). Adult beetles bored immature/mature cocoa beans through the pod husk, mucilage, and shell (Figure 5 and Figure 6a,b). The walls of galleries inside the beans were covered with whitish-grey fungal propagules (Figure 6c). Immature life stages (i.e., eggs, larvae, and pupae) were packed inside the galleries exhibiting a profuse whitish fungal mycelium (Figure 6d). The mean width of galleries located in pod husk, mucilage, and beans was 1.19 ± 1.15 mm (*n* = 30). However, the length of galleries in cocoa beans varied between 7.36–8.81 mm with a mean gallery length of 7.98 ± 0.45 mm (*n* = 30).

### 3.3. Associated Fungi Identification

The identification of the fungi associated with *X. crassiusculus* mycangia and galleries led to three different taxa.

**Figure 7:**
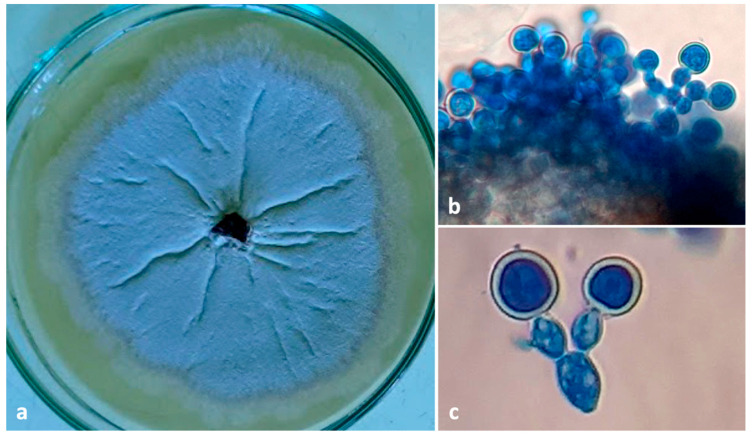
(**a**) Culture plate of *Ambrosiella roeperi*; (**b**) sporodochial mass *A. roeperi* isolated from mycangia; (**c**) branched conidiophore with terminal aleurioconidia.

A filamentous fungus with all the morphological features matching the descriptions of *Ambrosiella roeperi* by Harrington and McNew (2014) and Ceratocystidaceae given by [21] and [18] was consistently isolated from both beetle and galleries. Colonies of *A. roeperi* on PDA appeared brownish-grey on the media and whitish to vinaceous buff at the center after 6–7 d (Figure 7a) with a strong sweet odor. The underside of the colony appears dusky brown to black. Sporodochia and conidiophores with terminal aleurioconidia on distended conidiophore cells were observed (Figure 7b,c). The identity of *A. roeperi* was further confirmed by amplifying 580 bp in the ITS gene. The nucleotide sequences of the ITS gene were submitted to GenBank (GenBank accession No. OM438124-OM438125), which are 99.48% similar to *A. roeperi* (GenBank accession No. MK118927).

**Figure 8:**
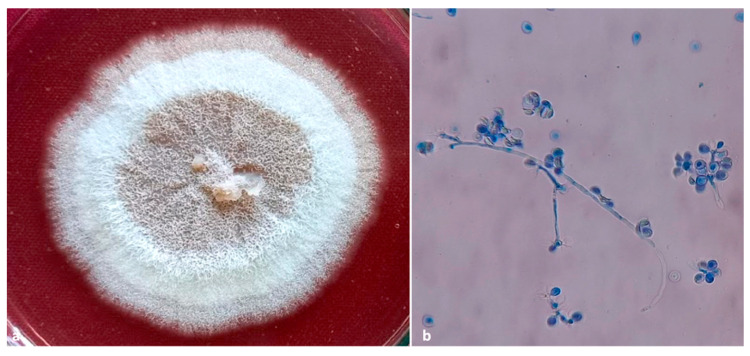
(**a**) culture plate of *Ambrosiozyma monospora*; (**b**) presence of globose asci in clusters at nodes of the ascophoric hyphae.

An ascomycetous yeast was also found with *A. roeperi* from mycangia of the beetles (*n* = 2) and insect galleries (*n* = 4) inside beans. The morphological characters of this yeast are similar to the descriptions of *Ambrosiozyma monospora* (Saito) in van der Walt (Saccharomycetales) [22]. Colonies of *A. monospora* on PDA appear whitish-grey at the periphery and greyish-brown at the center (Figure 8a). Microscopic characteristics of this yeast were observed as the presence of globose asci occurring in clusters at nodes of the ascophoric hyphae (Figure 8b). The identity of *A. monospora* was further confirmed by amplifying 700 bp of the ITS gene. The single representative nucleotide sequence of the ITS gene was submitted to GenBank (GenBank accession No. OM438126) and showed 99.57% similarity with *A. monospora* (GenBank accession No. KY101644, isolate CBS:5515).

The fungal isolate *Fusarium keratoplasticum* as described by Geiser et al. (Hypocreales) belonging to the *Fusarium solani* species complex was also isolated from insect galleries (*n* = 2) only located in the pod husk. The single representative nucleotide sequence of the *ITS* gene (accession number OM535898), *Tef1α* gene (accession number OM638602), and *RPBI* gene (accession number OM638603) was submitted to GenBank.

### 3.4. Choice Test Assay against Healthy and Mirid Affected Cocoa Pods

All female beetles (*n* = 60) from all replicates bored into mirid-infested pods within 24 h after being released in cages. Furthermore, all the beetles bored only through the dark circular lesions produced by the feeding activity of *H. theivora*.

Destructive sampling of all the pods after five days from the initial infestation documented the presence of adult beetles inside the beans. Only small circular entry holes were present inside the husk and mucilage, without any visible fungal garden (Figure 5a–d). However, all the active galleries (presence of adult/immature stages) were broad and oval with the presence of fungal propagules and located exclusively inside the cocoa beans (Figure 6c,d).

## 4. Discussion

Ambrosia beetles, including *X. crassiusculus,* generally colonize physiologically stressed or weakened plants [25]. Attacks are mostly concentrated on the woody tissues of the host plant and are mainly attributable to environmental stressors such as drought, flooding, freezing, and mechanical damage [26]; furthermore, plant pathogens can enhance or modify plant colonization patterns by scolytine beetles [27,28,29]. *Xylosandrus crassiusculus* usually displays xylomycetophagous habits, colonizing weak or stressed plants emitting ethanol [30,31]; however, it has been recently observed successfully adopting spermatophagous behavior on healthy immature areca nuts [18].

The results provided in the present study are not conclusive but suggest that *X. crassiusculus* is capable of successfully implementing spermatophagy in cocoa pods. Although the attacks of *X. crassiusculus* on cocoa pods were similar to those recently recorded on areca nuts [18], the modest number of plants attacked suggests that spermatophagy on cocoa pods is sporadic. It has been reported that Xyleborine beetles are capable of boring on unsuitable substrates, probably attracted by strong olfactory stimuli [32,33,34]. Therefore, it is possible that *X. crassiusculus* was misled by affected cocoa pods while looking for new hosts.

In this study, *H. theivora* played a key role in promoting the massive colonization of cocoa pods and specifically being responsible for the volatiles attracting *X. crassiusculus.* Our results clearly indicated that *X. crassiusculus* attacked only those cocoa pods damaged by *H. theivora,* and both lab and field observations showed that the pod entrance holes of *X. crassiusculus* matched almost perfectly with necrotic lesions caused by the piercing of the bug *H. theivora*. Most likely, these injured tissues release stress-related volatiles, including ethanol, similar to those used by *X. crassiusculus,* to spot physiologically stressed or mechanically injured trees [26,35,36] or suitable fruits, as already suggested in [18]. The fact that no woody parts of cocoa plants were attacked suggests that trees were not stressed, and the damaged cocoa pods were the only source of attraction for *X. crassiusculus*.

The *X. crassiusculus* adults penetrated the fruit through the meso and endocarp, the mucilaginous pulp, and settled its colonies inside the cocoa beans. In particular, the excavation of the brood chambers and the cultivation of symbiont fungi occurred exclusively inside cocoa beans, suggesting a high specificity in terms of the selection of the reproductive substrate by *X. crassiusculus*. This behavior and the spermatophagus habit presented the same modalities as reported by [18] for *Areca catechu* L., in which *X. crassiusculus* developed their brood chambers exclusively in the areca nut kernel.

The characterization of the microorganisms (fungi and yeasts) associated with *X. crassiusculus* and its galleries suggest that a particular combination of species and strains may allow the development of this xyleborine species in plant tissues other than wood.

*Ambrosiella roeperi* is the primary fungal mutualist of *X. crassiusculus,* and it constitutes the main food resource for the beetle and its larvae [21]. However, this fungus appears to be extremely adaptable, allowing *X. crassiusculus* to develop in a wide variety of host plants. Its ability to establish and proliferate would appear to be influenced by chemical features of the substrate, including the concentration of ethanol [31,36].

Beside a series of complex polysaccharides, cocoa beans contain up to 12 alcoholic compounds [37] whose presence and concentration could influence the development of *Ambrosiella*; therefore, detoxifying agents and microorganisms capable of metabolizing polysaccharides are probably required to guarantee the reproductive success of the beetle. This role could be played by the yeast *Ambrosiozyma monospora*, the second species of microorganism recorded in both mycangia and galleries. Although the role of yeasts in ambrosia beetle biology is not fully elucidated, members of the genus *Ambrosiozyma* are considered actual ambrosia yeasts [38] possibly able to detoxify plant phytochemicals, to provide nutrition for the beetles, and to regulate fungal growth by producing antagonistic metabolites that affect the establishment of filamentous fungi, entomopathogens, and opportunistic saprophytes [39,40,41]; in particular, *A. monospora* is known to be one of the best L-arabinose and D-xylose fermenting yeasts [42]. Arabinose is a major polysaccharide in the cell walls of cocoa beans [43] and *A. monospora* has the potential to ferment L-arabinose to ethanol in significant quantities [42]. These characteristics would suggest a possible function of *A. monospora* as a symbiont capable of facilitating *X. crassiusculus* colonization by degrading cocoa cell walls and enriching the substrate with ethanol to an optimal concentration for the proliferation of *A. roeperi*.

*Ambrosiozyma monospora* is reported as a fungal associate of *Xyleborus volvulus* (Fabricius, 1775) and *Xyleborus bispinatus* (Eichhoff, 1868) [44,45,46] and is here associated with *Xylosandrus crassiusculus* for the first time. The presence of low mycangial inoculum and insect galleries would be a possible reason for the non-detection of *A. monospora* in all the sampled *X. crassiusculus* adults and galleries.

*Fusarium keratoplasticum* was the third microorganism detected; however, it was recorded only from the entrance hole of two *X. crassiusculus* galleries. Rather than acting as direct vectors of *Fusarium* spp., *X. crassiusculus*, through mechanical injury, might facilitate the introduction of secondary pathogens that subsequently enhance their colonization success [47]. *Fusarium* spp. infections have been observed growing from the tunnel entrances and within the associated sap [14]. It is not known what role *F. keratoplasticum* plays in the biology of *X. crassiusculus*.

Cocoa beans are the basis for cocoa powder, butter, and chocolate production. Proper fermentation of harvested beans for up to seven days is of utmost importance [48]. The marketable quality of dry cocoa beans is dependent on the fermentation of fresh beans. Hence, an infestation of *X. crassiusculus* with fungal symbionts could interfere in the fermentation process of fresh cocoa beans and ultimately affect the marketable quality of dry cocoa beans.

In this regard, an efficient management protocol aimed at preventing the onset of beetle attacks and preventing and/or managing *Helopeltis* infestations could reduce the risk of cocoa bean contamination to a minimum, if not eliminate the problem.

## 5. Conclusions

The present study shows how little we still know about scolytine biology, even for some species in their native ranges. Furthermore, this recent observation of *X. crassiusculus* developing on cocoa beans indicates how ecologically plastic this beetle species is and suggests that this feeding behavior is directly linked to the fitness of its symbiotic fungi. In addition, positive interactions with native pests, such as *H. theivora,* might have important implications for the impact of *X. crassiusculus* on cocoa and other crops. Therefore, given the potential impacts of climate change and species niche shifting, it is necessary to further investigate the evolution of the spermatophagy of *X. crassiusculus* on the Indian subcontinent, to clarify the roles of its fungal symbionts through the beetle’s lifecycle, and to understand the role of other insect pests in enhancing *Xylosandrus* infestations on cultivated plants.

## Data Availability

The data that support the findings of this study are available from the corresponding author upon reasonable request.

## Supplementary Materials

The following supporting information can be downloaded at: https://www.mdpi.com/article/10.3390/insects13090809/s1, Table S1: details of the survey conducted on distribution of *X. crassiusculus* in cocoa in Karnataka.

## References

[1] Lahive F., Hadley P., Daymond A.J. (2019). The physiological responses of cacao to the environment and the implications for climate change resilience. A review. Agron. Sustain. Dev..

[2] Directorate of Cashewnut and Cocoa Development. https://dccd.gov.in/Content.aspx?mid=1072&tid=1.

[3] Beg M.S., Ahmad S., Jan K., Bashir K. (2017). Status, supply chain and processing of cocoa—A review. Trends Food Sci. Technol..

[4] Dand R. (2011). International Cocoa Trade.

[5] World Cocoa Foundation Report. https://www.worldcocoafoundation.org/wp-content/uploads/Cocoa-Market-Update-as-of-4-1-2014.pdf.

[6] Thube S.H., Mahapatro G.K., Mohan C., Pandian R.T.P., Apshara E., Jose C.T. (2019). Biology, feeding and oviposition preference of *Helopeltis theivora*, with notes on the differential distribution of species of the tea mosquito bug species complex across elevations. Anim. Biol..

[7] Entwistle P.F. (1972). Pests of Cocoa: Tropical Science Series.

[8] Mariau D. (1999). Integrated Pest Management of Tropical Perennial Crops.

[9] CPCRI (1993). Annual Report.

[10] Padi B. Prospects for the control of cocoa capsids—Alternatives to chemical control. Proceedings of the First International Cocoa Pests and Diseases Seminar.

[11] Muhamad R., Way M.J. (1995). Relationships between feeding habits and fecundity of *Helopeltis theivora* (Hemiptera: Miridae) on cocoa. Bull. Entomol. Res..

[12] Thube S.H., Saneera E.K., Prathibha P.S. (2016). Pests of Cocoa and their management. Cashew Cocoa J. Spec. Issue Cocoa.

[13] Saunders J.L. (1965). The *Xyleborus-Ceratocystis* complex of cacao. Cacao.

[14] Ranger C.M., Reding M.E., Schultz P.B., Oliver J.B., Frank S.D., Addesso K.M., Chong J.H., Sampson B., Werle C., Gill S. (2016). Biology, ecology, and management of non-native ambrosia beetles (Coleoptera: Curculionidae: Scolytinae) in ornamental plant nurseries. J. Integr. Pest Manag..

[15] Vega F.E., Infante F., Johnson A.J., Vega F.E., Hofstetter R.W. (2015). The genus *Hypothenemus*, with emphasis on *H. hampei*, the coffee berry borer. Bark Beetles: Biology and Ecology of Native and Invasive Species.

[16] Smith S.M., Beaver R.A., Cognato A.I., Hulcr J., Redford A.J. Southeast Asian Ambrosia Beetle ID. USDA APHIS Identification Technology Program (ITP) and Michigan State University. https://idtools.org/id/wbb/sea-ambrosia/.

[17] Thube S.H., Mohan C., Pandian R.T.P., Saneera E.K., Sannagoudra H.M., Hegde V., Chowdappa P. (2018). First record of the invasive neotropical ambrosia beetle *Euplatypus parallelus* (Fabricius, 1801) (Coleoptera: Curculionidae: Platypodinae) infesting arecanut in Karnataka, India. Coleopt. Bull..

[18] Thube S.H., Pandian T.P., Bhavishya A., Babu M., Josephrajkumar A., Chaithra M., Hegde V., Ruzzier E. (2022). *Xylosandrus crassiusculus* (Motschulsky) (Coleoptera: Curculionidae) and its fungal symbiont *Ambrosiella roeperi* associated with arecanut kernel decay in Karnataka, India. Insects.

[19] Sambrook J., Russell D.W. (2001). Molecular Cloning: A Laboratory Manual.

[20] Li Y., Ruan Y.Y., Stanley E.L., Skelton J., Hulcr J. (2019). Plasticity of mycangia in *Xylosandrus* ambrosia beetles. Insect Sci..

[21] Harrington T.C., McNew D., Chase M., Fraedrich S.W., Reed S.E. (2014). *Ambrosiella roeperi* sp. nov. is the mycangial symbiont of the granulate ambrosia beetle, *Xylosandrus crassiusculus*. Mycologia.

[22] Van der Walt J.P. (1972). The yeast *Ambrosiozyma* gen. nov. Ascomycetes. Mycopathol. Mycol. Appl..

[23] White T.J., Bruns T., Lee S., Taylor J., Innis M.A., Gelfand D.H., Sninsky J.J., White T.J. (1990). Amplification and direct sequencing of fungal ribosomal RNA genes for phylogenetics. PCR Protocols: A Guide to Methods and Applications.

[24] Pandian R., Thube S.H., Merin B., Pratibha V.H., Rajkumar M., Mhatre P.H., Hegde V. (2022). First record of *Fusarium falciforme* (FSSC 3 + 4) a relevant human pathogen causing root decay of arecanut, *Areca catechu* L. J. Plant Dis. Prot..

[25] Kühnholz S., Borden J.H., Uzunovic A. (2001). Secondary ambrosia beetles in apparently healthy trees: Adaptations, potential causes and suggested research. Integr. Pest Manag. Rev..

[26] Ranger C.M., Reding M.E., Addesso C., Ginzel M., Rassati D. (2021). Semiochemical-mediated host selection by *Xylosandrus* spp. ambrosia beetles (Coleoptera: Curculionidae) attacking horticultural tree crops: A review of basic and applied science. Can. Entomol..

[27] Rassati D., Contarini M., Ranger C.M., Cavaletto G., Rossini L., Speranza S., Faccoli M., Marini L. (2020). Fungal pathogen and ethanol affect host selection and colonization success in ambrosia beetles. Agric. For. Entomol..

[28] McPherson B.A., Mori S.R., Wood D.L., Storer A.J., Svihra P., Maggi Kelly N., Standiford R. (2005). Sudden oak death in California: Disease progression in oaks and tanoaks. For. Ecol. Manag..

[29] McPherson B.A., Erbilgin N., Wood D.L., Svihra P., Storer A.J., Standiford R.B. (2008). Attraction of ambrosia and bark beetles to coast live oaks infected by *Phytophthora ramorum*. Agric. For. Entomol..

[30] Chong J., Reid L., Williamson M. (2009). Distribution, host plants, and damage of the black twig borer, *Xylosandrus compactus* (Eichhoff), in South Carolina. J. Agric. Urban Entomol..

[31] Cavaletto G., Faccoli M., Ranger C.M., Rassati D. (2021). Ambrosia beetle response to ethanol concentration and host tree species. J. Appl. Entomol..

[32] Browne F.G. (1961). The biology of Malayan Scolytidae and Platypodidae. Malayan Forest Rec..

[33] Carlton C., Bayless V. (2011). A case of *Cnestus mutilatus* (Blandford) (Curculionidae: Scolytinae: Xyleborini) females damaging plastic fuel storage containers in Louisiana, USA. Coleopt. Bull..

[34] Wood S.L. (1982). The bark and ambrosia beetles of North and Central America (Coleoptera: Scolytidae), a taxonomic monograph. Great Basin Naturalist Memoirs.

[35] Ranger C.M., Reding M.E., Persad A.B., Herms D.A. (2010). Ability of stress-related volatiles to attract and induce attacks by *Xylosandrus germanus* and other ambrosia beetles (Coleoptera: Curculionidae, Scolytinae). Agric. For. Entomol..

[36] Ranger C.M., Schultz P.B., Frank S.D., Chong J.H., Michael E.R. (2015). Non-native ambrosia beetles as opportunistic exploiters of living but weakened trees. PLoS ONE.

[37] Qin X.W., Lai J.X., Tan L.H., Hao C.Y., Li F.P., He S.Z., Song Y.H. (2017). Characterization of volatile compounds in Criollo, Forastero, and Trinitario cocoa seeds (*Theobroma cacao* L.) in China. Int. J. Food Prop..

[38] Hulcr J., Stelinski L.L. (2017). The ambrosia symbiosis: From evolutionary ecology to practical management. Annu. Rev. Entomol..

[39] Davis T.S. (2015). The ecology of yeasts in the bark beetle holobiont: A century of research revisited. Microb. Ecol..

[40] Adams A.S., Six D.L., Adams S.M., Holben W.E. (2008). In vitro interactions between yeasts and bacteria and the fungal symbionts of the mountain pine beetle (*Dendroctonus ponderosae*). Microb. Ecol..

[41] Davis T.S., Hofstetter R.W., Foster J.T., Foote N.E., Keim P. (2011). Interactions between the yeast *Ogataea pini* and filamentous fungi associated with the western pine beetle. Microb. Ecol..

[42] Dien B.S., Kurtzman C.P., Saha B.C., Bothast R.J. (1996). Screening for L-arabinose fermenting yeasts. Appl. Biochem. Biotechnol..

[43] Redgwell R.J., Hansen C.E. (2000). Isolation and characterisation of cell wall polysaccharides from cocoa (*Theobroma cacao* L.) beans. Planta.

[44] Cruz L.F., Rocio S.A., Duran L.G., Menocal O., Garcia-Avila C.D.J., Carrillo D. (2018). Developmental biology of *Xyleborus bispinatus* (Coleoptera: Curculionidae) reared on an artificial medium and fungal cultivation of symbiotic fungi in the beetle’s galleries. Fungal Ecol..

[45] Menocal O., Cruz L.F., Kendra P.E., Crane J.H., Ploetz R.C., Carrillo D. (2017). Rearing *Xyleborus volvulus* (Coleoptera: Curculionidae) on media containing sawdust from avocado or silkbay, with or without *Raffaelea lauricola* (Ophiostomatales: Ophiostomataceae). Environ. Entomol..

[46] Inward D.J.G. (2020). Three new species of ambrosia beetles established in the UK illustrate unresolved risks of timber importation. J. Pest. Sci..

[47] Mayers C.G., McNew D.L., Harrington T.C., Roeper R.A., Fraedrich S.W., Biedermann P.H., Castrillo L.A., Reed S.E. (2015). Three genera in the Ceratocystidaceae are the respective symbionts of three independent lineages of ambrosia beetles with large, complex mycangia. Fungal Biol..

[48] Guzmán-Alvarez R.E., Márquez-Ramos J.G. (2021). Fermentation of cocoa beans. Fermentation-Processes, Benefits and Risks.

