# Supplementary material for: Xylosandrus crassiusculus (Motschulsky) on Cocoa Pods (Theobroma cacao L.): Matter of Bugs and Fungi
Source: Insects. 2022 Sep 5;13(9):809. doi: 10.3390/insects13090809 (PMC9506327; doi:10.3390/insects13090809)
Supplement: Supplementary file 1 [file insects-13-00809-s001.zip › insects-1837934-supplementary.pdf]

Table S1: details of the survey conducted on distribution of *X. crassiusculus* in cocoa in Karnataka

Village	Latitude	Longitude	Age of plantation (Year)	Infested plantation (%)
Murulya	12° 41' 17.717"	75° 24' 52.362"	13	Absent
Boonadka	12° 41' 57.541"	75° 36' 3.480"	9	Absent
Alankar	12° 46' 42.289"	75° 20' 54.181"	11	Absent
Jogogara	13° 32' 41.437"	75° 23' 45.535"	12	Absent
Khandya	13° 32' 52.566"	75° 25' 7.636"	7	Absent
Enekallu	12°40' 45.29"	75° 29' 32.913"	11	Absent
Periyadka	12°49' 18.905"	75° 17' 41.812"	9	Absent
Bantra	12°44' 31.884"	75° 30' 1.681"	12	Absent
Puttur	12°48' 31.849"	75° 16' 1.18"	15	1.45
Vittal	12° 46' 43.60"	75° 06' 58.60"	14	4.54
